# Anxiety and Coping Stress Strategies in Researchers During COVID-19 Pandemic

**DOI:** 10.3389/fpubh.2022.850376

**Published:** 2022-05-19

**Authors:** Patrícia Batista, Anabela Afonso, Manuel Lopes, César Fonseca, Patrícia Oliveira-Silva, Anabela Pereira, Lara Pinho

**Affiliations:** ^1^Universidade Católica Portuguesa, Research Centre for Human Development, Human Neurobehavioral Laboratory, Porto, Portugal; ^2^Universidade Católica Portuguesa, CBQF – Centro de Biotecnologia e Química Fina – Laboratório Associado, Escola Superior de Biotecnologia, Porto, Portugal; ^3^Department of Mathematics, School of Science and Technology, University of Évora, Évora, Portugal; ^4^Center for Research in Mathematics and Applications (CIMA), Institute for Advanced Studies and Research, Évora, Portugal; ^5^S. João de Deus School of Nursing, University of Évora, Évora, Portugal; ^6^Comprehensive Health Research Centre (CHRC), Évora, Portugal; ^7^Education and Psychology Department, Campus Universitário de Santiago, University of Aveiro, Aveiro, Portugal

**Keywords:** COVID-19, researchers, anxiety, depression, stress, fear, coping strategies

## Abstract

The current COVID-19 pandemic has affected the whole world, leading to changes in one's personal and working life. Researchers have undergone extensive changes in their roles, mainly in the area of health care, with research into the virus now the priority.

**Aim:**

To assess the anxiety, depression, stress, fears, and coping strategies of Portuguese researchers during the COVID-19 pandemic.

**Participants and Methods:**

A total of 243 researchers, with an average age of 37.9 ± 9.6, participated in an online questionnaire. The study was performed between 1 June 2021 and 11 August 2021. The questionnaire included depression, anxiety, and stress (DASS-21), fear of COVID-19 scale (FCV-19S), and coping inventory for stressful situations (CISS).

**Results:**

The findings suggest being female and younger seem to be related to more significant fears. Singles and younger researchers showed higher values of stress, depression, and anxiety. Research areas, such as medical and health sciences, presented higher levels in the DASS-21 depression and stress scale (*p* < 0.05). Also, the results showed a moderate or moderate strong significant positive linear relationship between the scales (*p* < 0.001): DASS-21 stress, DASS-21 anxiety, and DASS-21 depression (*r* > 0.70); CISS-21 emotional-oriented with DASS-21 stress (*r* = 0.683), DASS-21 depression (*r* = 0.622), and DASS-21 anxiety (*r* = 0.557); and emotional fear and cognitive fear (*r* = 0.652).

**Conclusion:**

The findings of this study support the growing concern for the psychological well-being of researchers and the need for intervention with more extensive and diverse studies.

## Introduction

In December 2019, new pneumonia caused by a virus (SARS-CoV-2) of the coronavirus family emerged. It is thought to have originated in China, in Wuhan, and quickly spread worldwide (1–3). In March 2020, the World Health Organization (WHO) declared the existence of a pandemic situation. At that time, SARS-CoV-2 was already one of the biggest challenges to world health (2). The increasing number of infections and death related to the COVID-19 disease led to increased concern by health organizations, governments, and society.

These concerns have been substantially exacerbated by extensive media coverage and continuous social media (mis)information, factors that generate fear, anxiety, social panic, and suicide risk (4–6). The need to know more about the disease and the virus, the need for scientific evidence to make decisions, the constant search for strategies, and methodologies to combat the problem have caused science to evolve at an unprecedented pace, namely in the field of vaccine development (7, 8).

At this moment, society renewed recognition of the role of science in fighting the pandemic. Across the world, most governments repeated in most press conferences: “We are following the science” (9). This is one step in the recognition that science/scientific knowledge is necessary for the prevention and search for solutions when we face contemporary challenges. However, the crucial steps are funding/economic investment into research projects and hiring human resources (10–12).

In the most several areas, these professionals who work for science and the increase in knowledge also had to readap not only in personal terms but also at the work level. While some research work can be done at home, such as article writing, scientific research, others require data collection, field presence, laboratory trials, and clinical trials (13). For instance, computational research and review studies about several thematic, such as rethinking psychology and the microbiota-gut-axis (14), may not have been much affected (15). However, much of research within the basic sciences involves laboratory work or clinical research, for example, studies that aimed to evaluate adverse event profiles of drugs in advanced prostate cancer and which require recruitment of participants for evaluation (16), were very much affected because they had to be suspended (15). These are two simple examples of scientific work of extreme importance but using different research methodologies.

Scientists do distinct work ranging from research, planning experiments, collecting and analyzing data, writing papers, writing fundraising proposals, teaching, clinical practice, administrative, and editorial activities. Not surprisingly, many studies have already shown that most of the pandemic-related decisions have magnified disparities among these researchers (13, 17–19). For instance, the research work and the time devoted to it were massively affected during the pandemic. Many researchers had to readapt their schedules and commitments, and in some cases, change their working methods because the access to field/laboratory work was restricted by confinement measures (13, 20–22). Many clinical trials were suspended due to the need for social isolation and multiple research groups felt the need to change their research projects and/or develop new ones, focusing on strategies to respond to the pandemic (13, 20). Teleworking and supporting children and dependents were other necessary readjustments (15, 23–25).

Although the pandemic affected the researchers' work in general, some researchers were more affected than others depending on their research areas, careers, and gender. There are already some publications in this sense, which report that the areas of biological sciences, biochemistry, and chemistry were more affected compared to the areas of mathematics and computer sciences (13). Similarly, studies have shown that early-career researchers (13, 26, 27) were also more conditioned by the pandemic, as well as the female gender (28, 29).

These labor and personal struggles in several areas are factors that increase the level of stress and anxiety and impact mental health significantly. However, few studies have been carried out at the level of this professional class, to understand the impact of the pandemic on the researchers' mental health, with particular emphasis on the anxiety during the lockdown (30).

Several studies have been exploring the anxiety of health professionals (31–35), academics (2, 36, 37), and the general population (38, 39). The levels of depression and anxiety were significantly higher during the outbreak and there was a need to study this topic. However, the concern with researchers is scarce (40–42) and it is urgent to cover this gap. Some studies, just prior to COVID-19, have been reported that researchers present high levels of stress (40, 43, 44). This shows that this problem existed even before COVID-19 and needs to be addressed.

On the other hand, in the attempt to resilience this problem it is necessary to implement adequate prevention or rehabilitation strategies. It is important to know positive and protective strategies to deal with this problem. Several studies have been carried out to develop and/or apply strategies to fill this gap in the population in general and in specific groups, in particular, but once again, the literature is scarce at the level of the researcher group. For example, in health professionals, several strategies were outlined, as include work-hour regulation programs, and the implementation of strategies to reduce the pressure of difficult decision-making (39). Some authors suggest interventions by the employer to improve the mental health of workers, such as providing the development of self-efficacy, resilience, promotion of social support, and guaranteeing quality and safe care (33, 45, 46).

Getting to know researchers better, motivating them, and promoting physical and mental well-being will bring benefits to their health, as well as to their role as researchers, contributing to the increase of scientific knowledge, fundamental for the improvement of the quality of life of our population. Thus, considering the health challenges for this understudied professional group, the aim of this study is to assess the levels of anxiety, depression, stress, fears, and coping strategies in Portuguese researchers during the COVID-19 pandemic. This knowledge is central to the development of intervention plans for these professionals, in the future.

## Methods

### Study Design and Participants

The target population was researchers working and living in Portugal. Inclusion criteria were to be a researcher in any scientific area and agree to participate in the online survey. This was a quantitative cross-sectional study that used a convenience sample (*n* = 243) of the Portuguese population recruited *via* e-mail (on professional networks). All participants gave their voluntary and informed consent, which was obtained electronically before recording any data from the participants.

### Measures

#### Data Collection

From 1 June 2021 to 11 August 2021, survey data were collected through an online questionnaire. The survey was constituted of 60 questions that took around 10 min to be completed. The questionnaire covered socio-demographic and professional information (e.g., age, sex, marital status, academic qualifications, research area, and professional activity), health-related data (general health perception and history of COVID-19 diagnosis), depression anxiety stress scale (DASS-21), fear of COVID-19 scale (FCV-19S) and coping inventory for stressful situations (CISS-21). Before the application, the questionnaire was validated by a senior researcher's panel, and then, it was transposed to Qualtrics software for final validation.

The online platform QualtricsTM software (Provo, UT, USA) was chosen because of the facilitation in the distribution and completion of surveys, according to the recommendations imposed on social distance. In addition, only the researchers directly involved in the study could access the data, thereby maintaining the confidentiality of research subjects and research data (47, 48).

This study was approved by the ethical committee, and data confidentiality was ensured by assigning a code to each participant. No identifiable data were collected from the participant.

#### Depression Anxiety Stress Scale (DASS-21)

The DASS-21 was a scale developed to explore the symptoms of depression, anxiety, and stress. In this study, we used the scale validated for the Portuguese population (49). The DASS-21 instrument comprises 7-item for each subscale. The responses were collected on a 4-point scale of severity/frequency that assesses the extent to which the individual experienced each state in the previous week.

#### Fear of COVID-19 Scale (FCV-19S)

The FCV-19S was developed with the intent to identify and early intervene, psychologically, in people with high values of fear of COVID-19 (50). Ahorsu et al. (50) have proposed this scale, with 7-items, that assesses distinct physiological reactions of fears related to COVID-19. In this study, we used the Portuguese version of the Coronavirus Anxiety Scale (CAS) (51).

#### Coping Inventory for Stressful Situations (CISS-21)

The CISS-21 was developed by (52) by a psychometrically valid and reliable self-reporting instrument to identify and assess coping skills (51, 53). There are two versions (21-items and 48-items), but the shorter version has been the most widely used (51, 53). In this specific case, we use the Portuguese version already validated by Pereira and Queirós (54).

### Statistical Analysis

Descriptive statistics were used to describe the study sample. The Pearson linear correlation was used to assess the linear correlation between age and scale, as well as between scales. The Shapiro–Wilk test was used to assess normality. The Levene test was used to assess variance homogeneity. The *t*-test was used to assess significant differences in scales by gender or type of contract. The Wilcoxon Mann–Whitney was used when the normality assumption was violated. To compare the scales by marital status or research area, the analysis of variance was used: the *F* test when both normality and homoscedasticity assumptions were verified, the Kruskal–Wallis test when only normality assumption was violated, or the Games–Howell test when the assumption of homogeneity of variances was violated.

Multivariate linear regression analyses were performed using the scores of the questionnaires as dependent variables, and gender, age, marital status, type of contract, and research area as the exploratory variables. These models allowed us to assess associations and check for confounders. It was used the forward and backward methods to select the variables. Normality and homoscedasticity assumptions were checked.

R program version 4.0.4 (R Core Team, Austria) for Windows was used to perform the statistical analyses. A significance level of 0.05.

## Results

### Sociodemographic and Health Characteristics

The sample used consisted of 243 participants, 69.5% female. The participants' age ranged between 21 and 72, being an average age of 37.9 ± 9.6 years. When analyzing the professional activity, 40.8% presented a contract with the institution/center of research, 44.1% presented no contract (research fellowship), and 15.2% answered “other situation.” The study included participants from various research areas, with the majority being in the “Natural and Agricultural Sciences” (33.7%) and the “Social Sciences” (21.8%).

Most of the participants perceive their health as good (67.1%) and 7.4% have been infected with COVID-19.

Table 1 shows the sociodemographic and health characteristics of the sample.

**Table 1:** Sociodemographic and health characteristics.

**Variável**	**Categorias**	***n***	**%**
Sex	Male	74	30.5
	Female	169	69.5
Marital status	Single	122	50.2
	Non-marital partnership	42	17.3
	Married	68	28.0
	Widower	3	1.2
	Separated/ divorced	8	3.3
Academic Qualifications	Undergraduate	10	4.1
	Master's Degree	110	45.3
	PhD	123	50.6
Type of contract	Research fellow	144	59.3
	Researcher with contract	99	40.7
Research Area	Medical and Health Sciences	27	11.1
	Exact Sciences	18	7.4
	Natural and Agricultural Sciences	82	33.7
	Engineering and Technology	30	12.3
	Social Sciences	53	21.8
	Humanities	16	6.6
	Other	17	7.0
General health perception	Poor/Low	21	8.6
	Good	163	67.1
	Very good	59	24.3
Has been/is infected with COVID-19	No	225	92.6
	Yes	18	7.4

**Figure 1:**
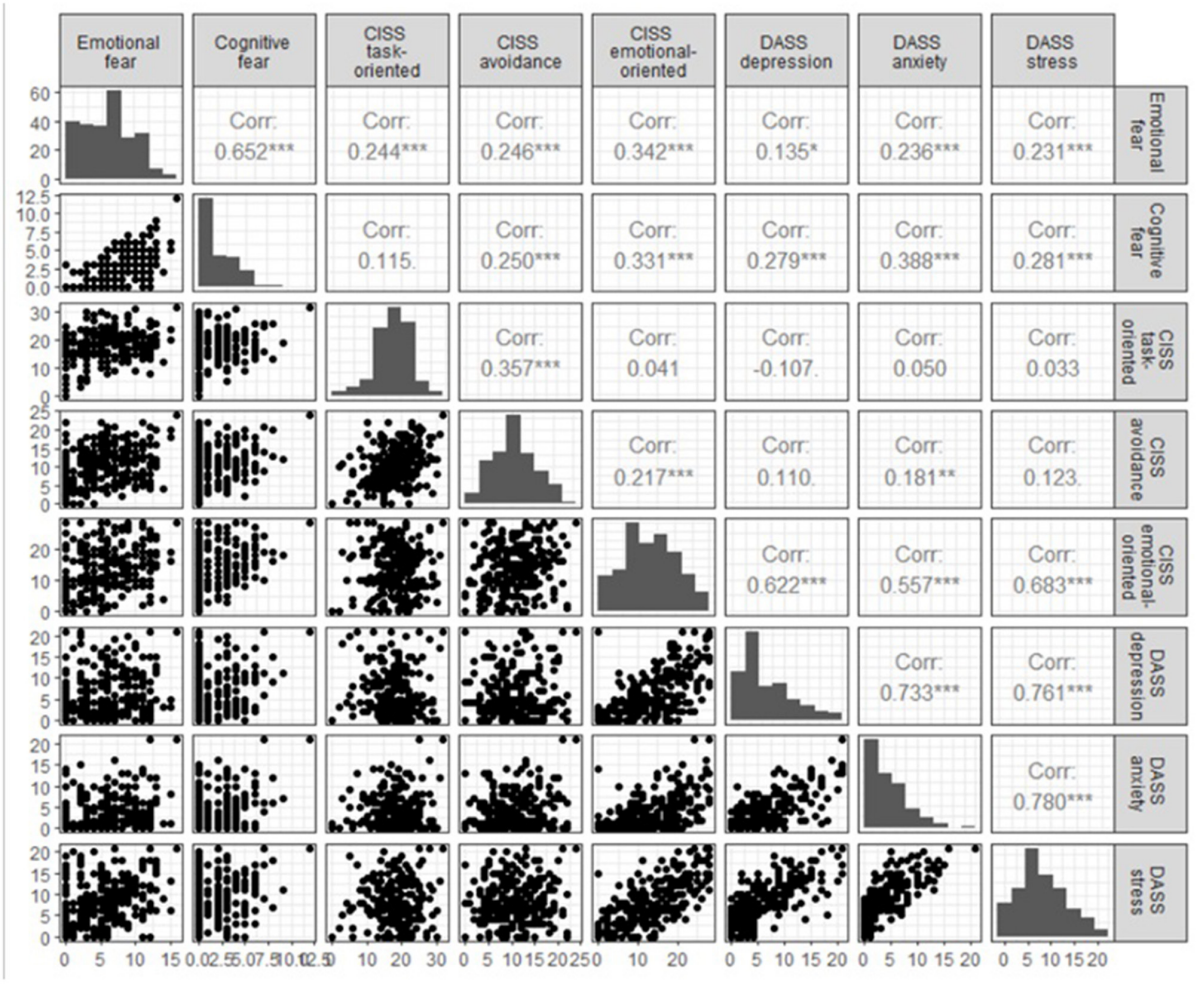
Empirical distribution of scales and Pearson's linear correlation coefficient between the scales *p < 0.1, **p < 0.05, ***p < 0.001.

When the scales selected for this study were analyzed, the low values stand out for the cognitive fear scale [Med = 0, IQR = (0, 3)], DASS-21 depression [Med = 4, IQR = (2, 9)], and DASS-21 anxiety [Med = 3, IQR = (1, 6)] (Figure 1). In the CISS-21 task-oriented (18.13 ± 5.67) and CISS-21 avoidance (10.58 ± 5.10) scales, intermediate values predominate. In the CISS-21 emotional-oriented (13.47 ± 7.39), emotional fear (5.95 ± 3.98), and DASS-21 stress (8.16 ± 5.09) scales there is great heterogeneity in the values observed. On the CISS-21 emotional-oriented scale there appears to be a similar frequency of responses across the range of possible values (uniform distribution).

The results showed a moderate or moderate strong significant positive linear relationship between the scales (*p* < 0.001, Figure 1):

- DASS-21 stress, DASS-21 anxiety, and DASS-21 depression (all *r* > 0.70);
- CISS-21 emotional-oriented with DASS-21 stress (*r* = 0.683), DASS-21 depression (*r* = 0.622), and DASS-21 anxiety (*r* = 0.557),
- Emotional fear and cognitive fear (*r* = 0.652).

### Analysis of Scale by Sociodemographic Characteristics

The differences between the scales and some variables, such as gender, age, professional activity, and research area, were studied.

#### Genders

Significant differences were only detected on the emotional fear scale between women and men (*W* = 5,160, *p* = 0.030); women [Med = 6, IQR = (3, 9)] had higher values than men [Med = 4.5, IQR = (2, 8)]. In the remaining scales, there were no significant differences between genders (*p* > 0.05).

#### Age

When age and scales were compared, there was a significant but weak negative linear relationship between age and the scales CISS-21 emotional-oriented, DASS-21 depression, DASS-21 anxiety, and DASS-21 stress (Table 2, *p* < 0.001). These data were indicators of the existence of a tendency for the higher values of these scales to be associated with younger researchers and for the lower values of these scales to be associated with older researchers.

**Table 2:** Pearson's correlation coefficient (*r*), and *p*-value (*p*), between age and scales.

**Scale**	**R**	***P***
Emotional fear	−0.080	0.213
Cognitive fear	−0.171	0.008
**DASS-21**		
Depression	−0.336	<0.001
Anxiety	−0.374	<0.001
Stress	−0.340	<0.001
**CISS-21**		
Task-oriented	0.051	0.428
Avoidance	−0.161	0.012
Emotion-oriented	−0.352	<0.001

The negative linear relationship between age and the cognitive fear and CISS-21 avoidance scales, although significant, is almost insignificant.

#### Marital Status

For the marital status analysis, the widowed and separated/divorced categories were joined, since there are only three widowers. We detected that cognitive fear, emotional-oriented CISS-21, and all the DASS-21 scales differ significantly between marital status (all *p* < 0.05, Table 3). Single people had higher values than married people on all these scales (all *p* < 0.05).

**Table 3:** Median (1st quartile, 3rd quartile), or mean and standard deviation, for each scale by marital status of the researchers and *p*-value from analysis of variance [^(1)^parametric ANOVA, ^(2)^Kruskal–Wallis test, ^(3)^ Games–Howell test].

**Scale**	**Single**	**Maried**	**Non-marital partnership**	**Separated/divorced/widowed**	***p***
Emotional fear	6 (3, 9)	5 (3, 8.25)	5 (3, 10)	5 (3, 7.5)	0.546^(2)^
Cognitive fear	1^b^ (0, 3)	0^b^ (0, 2)	2^ab^ (0, 3)	0^ab^ (0, 3)	^(3)^
CISS task-oriented	19 (15, 22)	19 (15.75, 22)	18 (14.25, 21)	17 (16, 19.5)	0.806^(2)^
CISS avoidance	11.14 (5.21)	10.06 (4.47)	10.17 (5.21)	9.27 (6.84)	0.367^(1)^
CISS emotional-oriented	15^b^ (9, 20)	10^a^ (7, 15.25)	15^ab^ (9, 19.75)	12^ab^ (5.5, 16)	0.001^(2)^
DASS depression	6.5^b^ (3, 11)^a^	3^a^ (1, 5.25)	4^ab^ (2, 9)	4^ab^ (2,5)	0.001^(2)^
DASS anxiety	4^b^ (1, 6)	3.5^a^ (1, 6.75)	3.5^ab^ (1, 6.75)	0^a^ (0, 3)	0.001^(2)^
DASS stress	9^b^ (6, 13)	6^a^ (3.75, 8.25)	7^ab^ (6, 11)	4^a^ (3, 8.5)	0.001^(2)^

*Medians or means not sharing superscript letters, in the same row, differ significantly at p < 0.05 as indicated by the post-hoc test*.

#### Type of Contract

No significant differences were found on any scale by type of contract of the researchers (all *p* > 0.05).

#### Research Area

**Figure 2:**
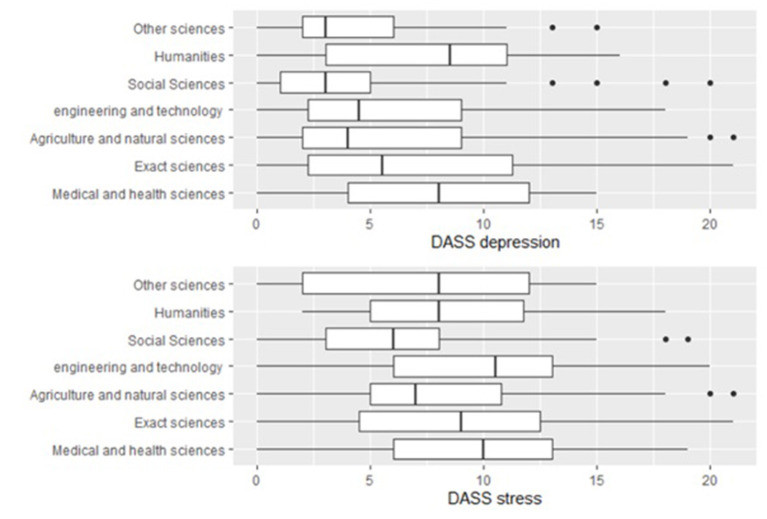
Empirical distribution of DASS-21 depression and DASS-21 stress scales by research area of the researchers.

There were significant differences in DASS-21 depression (*p* = 0.020) and DASS-21 stress (*p* = 0.042) scales between research areas (Figure 2). Researchers in the medical and health sciences had higher scores than those in the social sciences on the DASS-21 depression scale (*p* < 0.1). The multiple comparisons test did not detect which pairs of research areas significantly differed in the DASS-21 stress scale, but by the graphical analysis, researchers in the social sciences area seem to have lower values than those in other areas.

All the adjusted models for the several scores of the questionnaires allowed us to check the inexistence of confounders in most of the bivariate analyses presented in the previous sections on the emotional fear scale. However, the explanation power of the adjusted models was small (in all, $R_{\text{Adj}}^{2}$ < 0.2). The adjusted models for scores in emotional fear and CISS task-oriented did not fit the data. Older researchers had significantly lower scores in cognitive fear, CISS avoidance, CISS emotional-oriented, DASS depression, DASS anxiety, and DASS stress. The multivariate models revealed that women had significantly lower scores than men only in DASS depression (*b* = −0.362, *p* = 0.006). Also, researchers in exact sciences (*b* = −0.811, *p* = 0.021) and in agriculture and natural sciences (*b* = −0.585, *p* = 0.020) had significantly lower scores in cognitive fear than researchers in medical and health sciences.

## Discussion

This study seeks to understand the anxiety, stress, and depression researchers' perception during the pandemic period and the coping strategies that they were developed.

### Sociodemographic and Professional Characteristics

Regarding sex, differences are only observed in the emotional fear scale where women have higher values than men. Another author concludes that the higher fear reported by female gender can be explained by their higher sensitivity to stress when compared to the male gender (55). However, in our study, there are no differences in anxiety, stress, and depression between the sexes. These results are not consistent with most studies that report that women have higher levels of stress, anxiety, and depression (33, 55–57). These results may be due to having a sample of only researchers who may have a different response to these variables. It is important to note that regarding gender balance, women tend to be overrepresented in this profession as well as among such frontline service workers (58).

Younger researchers showed higher values of stress, depression, anxiety, and fears related to COVID-19 when compared to older researchers. Studies in the general population support these results by confirming that younger age groups are more vulnerable to symptoms of stress, depression, and anxiety (59, 60). As well as, when analyzing the fear toward COVID-19, the older researchers showed lower levels (55). However, it may be that older people may consider that they have little to lose as they have already had relatively long lives and had a stable labor situation. For their part, the younger people are worried about the future consequences and economic challenges caused by the pandemic, as they are the most affected by their employment stability, may watch and listen to much more negative news on social media (2, 61, 62). Nevertheless, additional evidence is needed to examine such speculation.

Single participants had higher scores of stress, depression, and anxiety than those who are married. Other studies have obtained similar results (59). Studies suggested that being married can be a protective factor for stress and anxiety (63).

Researchers in the medical and health sciences have higher levels of depression than those in the social sciences. Although we do not have identical studies with researchers from different fields to compare these results, several studies indicate the high prevalence of stress, anxiety, and depression in health care workers (64). Medical and health sciences researchers have had to change their research projects to give priority to pandemic-related research. Also, being their field, they are more awake to the pandemic health consequences, so these factors may be contributing to higher levels of depression. Social science researchers also have lower stress scores than researchers from other areas. Perhaps researchers in the social sciences are more prepared for changes in society, since they study social and collective behaviors, and this is the reason for lower stress levels. However, more studies are needed to draw conclusions.

### Anxiety, Stress, and Depression

In our study, analyzing the results of the DASS-21, we found that stress is the dominion with the highest mean (8.16 ± 5.09), followed by depression (6.01 ± 5.37), and anxiety (3.88 ± 4.09). These results are like a study in an Indian population with respect to the order of severity of the domains (65). However, the Indian study obtained higher values for the 31 researchers in the sample in all domains: stress (14.71 ± 9.89), depression (10.65 ± 8.72), and anxiety (9.81 ± 6.88).

### Coping Stress Strategies

The results showed that there is a significantly positive and moderately linear relationship between the anxiety levels and emotional-oriented coping strategies, i.e., general researchers with low (/high) anxiety values also have low (/high) emotional-oriented coping strategies. However, there is no significant linear relationship between the anxiety levels, and the task-oriented and avoidance coping strategies. These results corroborate another study that showed that depressive symptoms were positively correlated with emotional coping (66). We also verified that the stress levels are significantly positively and moderately linearly related to the emotional-oriented coping strategies, but it is not linearly related to the task-oriented and avoidance coping strategies. The depression levels are significantly related in a positive and moderate linear fashion with the emotional-oriented coping strategies and in a very weak negative linear fashion with the task-oriented coping strategies, but it is not linearly related to the avoidance coping strategies.

The task-oriented coping strategies were not supported but the relationship between the use of the emotional-oriented coping strategies was found. Although some studies report that emotion-focused and problem-focused strategies play role in reducing and increasing mental health (67), the unexpected event of COVID-19 pandemic can be may have triggered a more intense emotional response, indicating the need for further studies on this pandemic. But, their use can be inappropriate (66).

In this study, we did not find the results shown in other studies that showed that people that experienced psychological distress who used more task coping strategies experienced low levels of depression, anxiety, and stress (68).

The cognitive and emotional fears of COVID-19 pandemic situations also influence coping strategies or defensive mechanisms (69). In Huang and collaborators' study, it was found that fears were significantly positively related to problem-focused coping and emotion-focused coping. Therefore, the more problem-focused coping, the more fear (46). When analyzing the FCV-19S scale the data by emotional fear scale showed significantly related in a very weak positive linear way with CISS-21 emotional-oriented, task-oriented, and avoidance domains. On the order hand, the cognitive fear scale is significantly related in a very weak positive linear fashion to the emotional-oriented and avoidance coping strategies. There is no significant linear relationship between cognitive fear and task-oriented coping strategies.

### Limitations

This study presents some limitations, such as the cross-sectional nature of the study, which conditioned the monitoring of the effects and strategies adopted. Longitudinal studies are needed. Also, the methodology adopted, an online survey, may contribute to non-response bias in the study results. On the other hand, we do not know how many researchers there are in Portugal, because there are several contracting modalities, and many researchers are not in the career and presenting research grants (without contractual ties). So, it was not possible to calculate the sample size to ensure that the sample was representative.

## Conclusion

The findings of this study support the growing concern for the psychological well-being of researchers and the need for intervention. Being a female seems to be related to greater fears. Research areas, such as medical and health sciences, presented higher depression and stress levels. Also, significant differences were found between depression and emotional-oriented coping strategies, and the type of contract. The anxiety, depression, and stress levels were significantly related positively to emotional-oriented coping strategies.

This study intended to assess the levels of anxiety, depression, stress, fears, and coping strategies in Portuguese researchers during the COVID-19 pandemic. There is a gap in the literature in terms of scientific studies on these professionals, and this knowledge is central to the development of intervention plans for these professionals, in the future. However, this study suggests more extensive and diverse studies on the improvement of mental health and the reduction of anxiety/depression and stress in researchers. It is fundamental to investigate and intervene to promote the health of these professionals and their work performance, highlighting the importance of coping strategies. It is important to prioritize essential competencies, set goals, and coping strategies that increase health and performance.

## Data Availability Statement

The raw data supporting the conclusions of this article will be made available by the authors, without undue reservation.

## Ethics Statement

The studies involving human participants were reviewed and approved by Comissão de Ética da Universidade de Évora. The patients/participants provided their written informed consent to participate in this study.

## Author Contributions

PB, LP, and AP were responsible for the concept and design of the study, interpretation of results, writing, and critical review of the manuscript. PB and LP were responsible for data collection and analysis and writing—the original draft. PB, LP, and CF were responsible for the interpretation of the results. AA was responsible for statistical analysis. AP, PO-S, and ML were responsible for writing, reviewing, and editing the manuscript. All authors contributed to the article and approved the submitted version.

## Funding

The present publication was funded by Fundação Ciência e Tecnologia, IP national support through CHRC (UIDP/04923/2020). This work was partially supported by CEDH, through the Project UIDB/04872/2020 of the Fundação para a Ciência e a Tecnologia, Portugal. Also, the CIMA was supported by the Fundação para a Ciência e a Tecnologia, project UID/04674/2020.

## Conflict of Interest

The authors declare that the research was conducted in the absence of any commercial or financial relationships that could be construed as a potential conflict of interest.

## Publisher's Note

All claims expressed in this article are solely those of the authors and do not necessarily represent those of their affiliated organizations, or those of the publisher, the editors and the reviewers. Any product that may be evaluated in this article, or claim that may be made by its manufacturer, is not guaranteed or endorsed by the publisher.
